# High-altitude HEMS missions—a retrospective analysis of 3,564 air rescue missions conducted between 2011 and 2021

**DOI:** 10.1186/s13049-025-01419-x

**Published:** 2025-05-30

**Authors:** Eva Klocker, Lea Wienandts, Dario Josi, Simon Rauch, Roland Albrecht, Jürgen Knapp, Urs Pietsch

**Affiliations:** 1 Department of Anaesthesiology, Rescue and Pain Medicine, HOCH Health Ostschweiz St. Gallen, St. Gallen, Switzerland; 2 Department of Internal Medicine, HOCH Health Ostschweiz Altstaetten, Altstaetten, Switzerland; 3 University Clinic for Anaesthesiology and Pain Medicine, University Hospital Bern, Bern, Switzerland; 4 Swiss Air Ambulance (Rega), Zurich, Switzerland; 5 Aquatic Ecology and Evolution, Institute of Ecology and Evolution, University of Bern, Bern, Switzerland; 6 Department of Fish Ecology and Evolution, EAWAG, Swiss Federal Institute for Aquatic Science and Technology, Kastanienbaum, Switzerland; 7 Department of Anaesthesia and Intensive Care Medicine, Hospital Merano, Meran, Italy; 8 Institute of Mountain Emergency Medicine, Eurac Research, Bozen, Italy; 9 Division of Perioperative Intensive Care Medicine, HOCH Health Ostschweiz St. Gallen, St. Gallen, Switzerland; 10 Department of Emergency Medicine, Inselspital, University Hospital Bern, Bern, Switzerland

**Keywords:** HEMS, High altitude, Mountain medicine, Alpine rescue, On-scene time

## Abstract

**Background:**

Mountain sport activities are being practiced by an increasing number of people: The number of tourists visiting altitudes greater than 2,500 m above sea level in the Alps has been estimated at around 40 million people per year. For this reason, however, the number of emergencies in remote areas, which can be reached most rapidly by helicopter, has also increased.

**Methods:**

We retrospectively reviewed all rescue missions conducted by the Swiss Air Ambulance (Rega) in the period 2011–2021 that were carried out at an altitude of more than 2,500 m above sea level. Demographic and epidemiological data, medical measures implemented on scene, and the on-scene time were then analyzed for both trauma and non-trauma patients. Patients were categorized based on the National Advisory Committee for Aeronautics (NACA) score into non-injured (NACA 0), minor injured (NACA 0–3), seriously injured (NACA 4–6), deceased during mission (NACA 7), and already deceased on arrival of the HEMS team.

**Results:**

A total of 3,564 rescue missions were analyzed. Of the patients, 66.8% were male and the vast majority (88.4%) were adults. In terms of injury level, 88.1% of the patients were minor injured, with an NACA score of 0–3, while 9.4% were seriously injured, with a score of 4–6. Patients who died in scene (NACA 7) accounted for 2.5% of cases. We observed a significant increase in the number of minor injured patients with traumatic injuries over the period of observation. Factors that significantly influenced the on-scene time included the NACA score, hoist missions, and traumatic injuries in summer.

**Conclusion:**

Over the last ten years, the number of HEMS missions conducted at more than 2,500 m above sea level with non-injured and slightly injured patients has increased. The large number of HEMS missions with uninjured patients are of a preventive nature. Only around 9% of all rescue missions involved the medical treatment and rescue of seriously injured patients who required advanced medical interventions.

**Trial registration:**

Ethics approval and consent to participate BASEC Nr. Req202200189.

**Supplementary Information:**

The online version contains supplementary material available at 10.1186/s13049-025-01419-x.

## Background

The popularity of mountain sports is increasing, with a growing number of people participating in recreational activities at altitudes greater than 2,500 m above mean sea level (amsl.), which is classified as “high altitude” according to international standards [1]. In recent years, there has been a significant increase in hiking, climbing, backcountry skiing, and mountaineering [2, 3], with. around 40 million tourists engaging in these high-altitude experiences in Europe every year [1, 2].

Concomitantly, there has been an increase in the number of emergencies occurring in remote mountainous locations that can often only be accessed within a reasonable timeframe by helicopter. Helicopter Emergency Medical Services (HEMS) therefore play a crucial role in this challenging environment, providing access to high-quality emergency medical services, with the specific goal of reducing the treatment-free interval for time-critical injuries such as major trauma [3–5]. It is also of critical importance to be able to reach minor to moderately injured patients in remote regions in a timely manner, given the potential risks of hypothermia or patient exhaustion in challenging environments [6, 7]. However, there is currently little literature available on HEMS missions above 2,500 amsl.

## Methods

The aim of this study was to investigate the characteristics of high-altitude HEMS missions and describe the severity of injuries. The study consisted of a retrospective analysis of prospectively collected data from the Swiss Air Ambulance (Rega). All primary rescue missions conducted at an altitude above 2,500 amsl. from January 1, 2011, to December 31, 2021, were included.

The Ethics Committee of Eastern Switzerland (EKOS 2022 − 00189 St. Gallen, Switzerland) approved this retrospective observational study. The study was conducted in line with the Declaration of Helsinki and the Swiss Act on Human Research. Our reporting conforms to the applicable STROBE guidelines.

### Setting

The Swiss Air Ambulance REGA is the largest of three physician-staffed HEMS organizations in Switzerland. All REGA helicopters are equipped with a hoist. The HEMS crew includes a pilot, a flight paramedic who is also trained as a hoist operator, and a physician. Most physicians have completed additional training in mountain emergency medicine. Since the paramedics serve as hoist operators, they may not be available for patient care during a hoist mission [8].

### Study design and data collection

The data for this study was taken from the electronic master file of all HEMS missions carried out by Rega since 2011 (SAP database). It comprises demographic patient data (e.g., age and sex), mission data (e.g., mission GPS coordinates, alarm reason, type of activity, type of accident), and basic medical data (e.g., diagnoses, interventions). Injury severity was classified using the National Advisory Committee for Aeronautics (NACA) score.

For all patients with NACA ≥ 4, vital parameters, medical interventions, and handwritten notes concerning the injury mechanism and clinical findings were retrieved. No patient-identifying variables were collected.

### Data outline

The NACA scores were organized into four groups: non-injured patients (NACA 0), minor injured patients (NACA 1–3), severely injured patients (NACA 4–6), and patients who died on-site but underwent medical procedures (NACA 7). Patients who died prior to the arrival of the HEMS team were categorized separately as “dead before arrival”, as the primary objective in such cases was to recover the body. Demographic data and basic medical measures, such as analgesia and intravenous access, were analyzed across all NACA groups. A detailed analysis of injury patterns, on-scene measures, and factors influencing on-scene time was specifically conducted for the NACA 4–6 group.

The winter season spanned from December to April and the summer season from May to November. The aeronautical night was defined as extending from 30 min after sunset to 30 min before sunrise. A remote area was defined as a place that could not be reached by a means of regular transport. Injuries were classified by type into non-trauma and trauma. The Glasgow Coma Score (GCS) was also grouped into four categories: no impairment of consciousness (15–14 points), mild impairment (13–12 points), moderate impairment (11–9 points), and severe impairment (8–3 points), aligning with the severity assessment for traumatic brain injuries.

Medical interventions on scene were subdivided into basic measures and additional measures (these were only assessed for severely injured patients with NACA ≥ 4) as shown in Tables 1 and 2 in the Appendix.

The on-scene time was defined as the period between the helicopter’s arrival at the accident site and its departure from the scene.

### Statistical analyses

The data were analyzed using R version 4.2.0, and a linear regression model was fitted to predict on-scene time. The predictors included in the model comprised: altitude, location (accessible/remote), daytime (day/night), season (summer/winter), hoist (yes/no), NACA score [4–6], and trauma (yes/no). The interaction between trauma (yes/no) and season (summer/winter) was also analyzed using the model. Model summary statistics and diagnostics were then examined to evaluate the goodness of fit.

The emmeans package (version 1.8.8) in R was used to calculate estimated marginal means (or least squares means) for the levels of the predictor variables. These estimated marginal means represent the average response variable (on-scene time) for each level, while accounting for the other predictors in the model. Post-hoc assessments were performed using TukeyHSD tests. The coefplot function from the coefplot package (version 1.2.8) was used to visualize the estimated coefficients (without interaction), enabling a clear and concise representation of the model parameters. The resulting coefficient plot visually presents the magnitude and direction of the effects of each predictor variable on the on-scene time, aiding in the interpretation and communication of the model results.

## Results

**Fig. 1:**
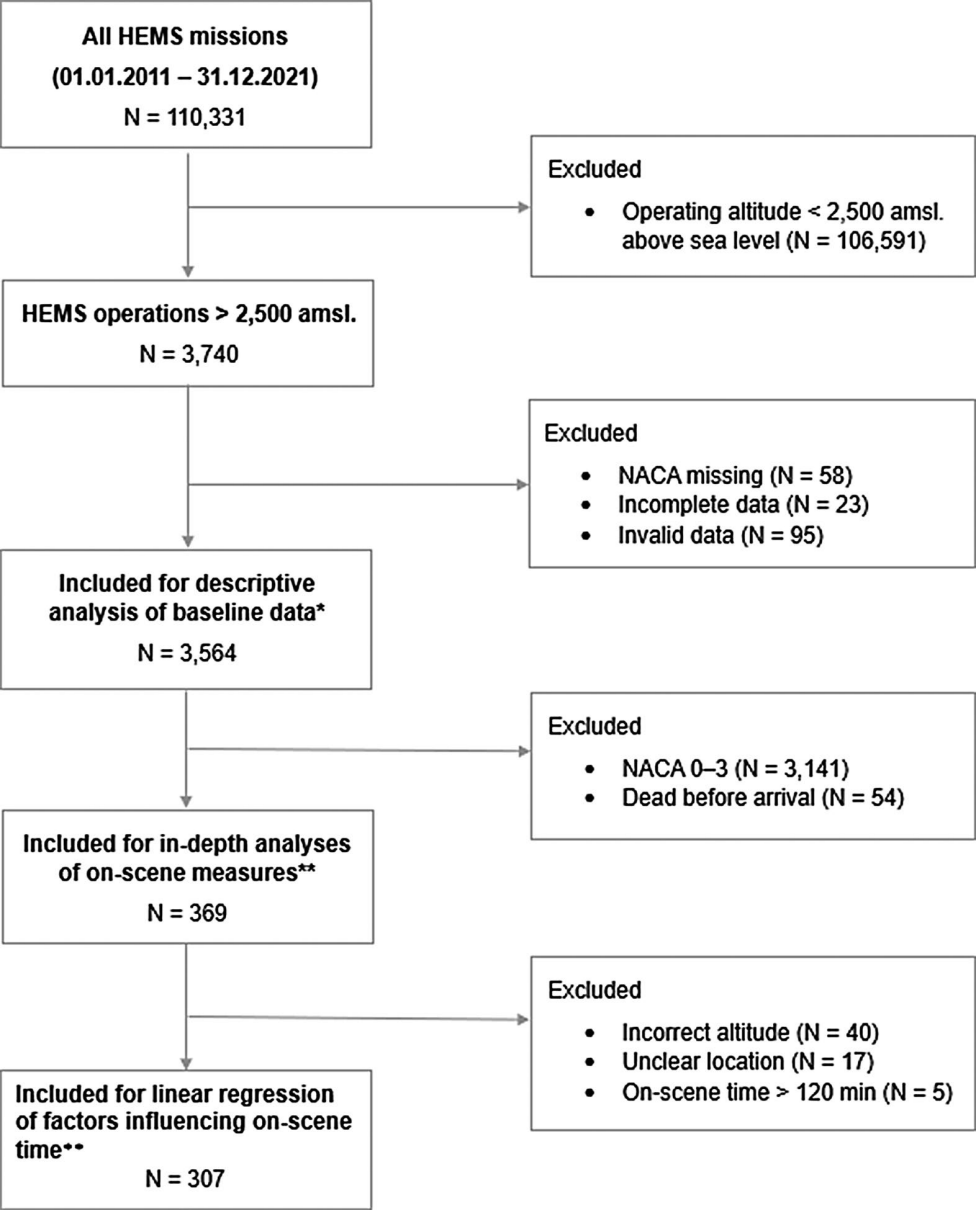
HEMS missions between 2011 and 2021 conducted above 2,500 amsl with the excluded data. * Data from the SAP database; ** Data from the medical reports of the HEMS team

We screened 110,331 missions, of which 3,564 were used in the final analysis (Fig. 1).

Patient and mission characteristics are summarized in Tables 1, 2 and 3.

**Table 1:**
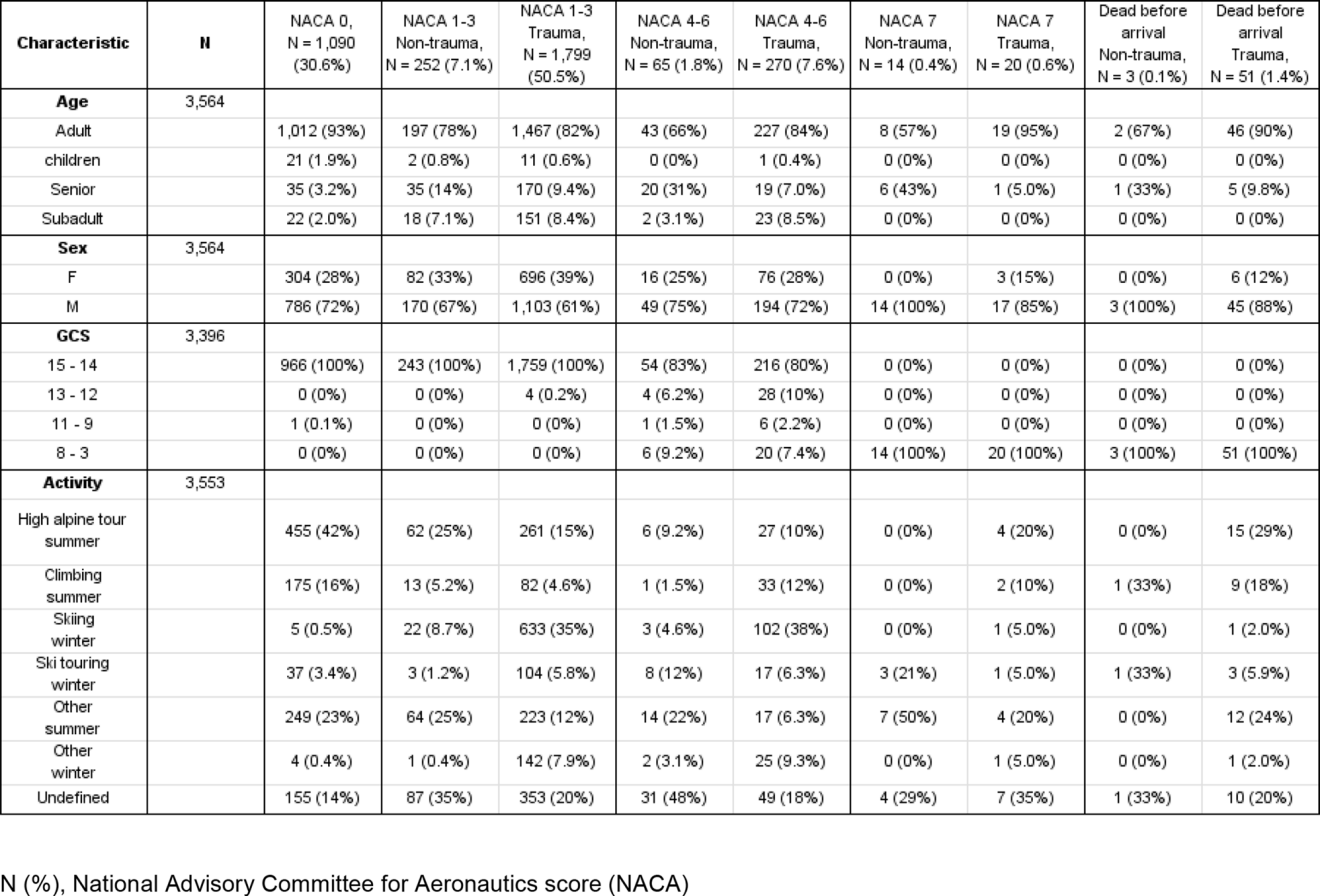
Baseline patient data of HEMS missions conducted above 2,500 amsl. (2011–2021)

**Table 2:**
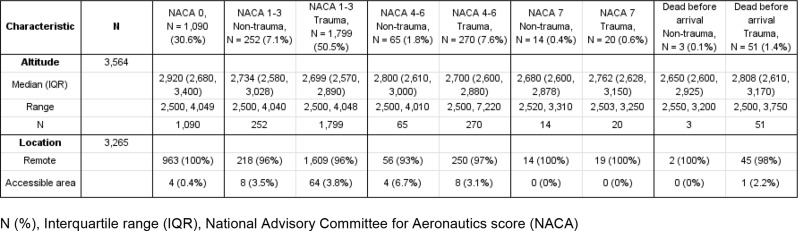
Baseline data on location and altitude of HEMS missions conducted above 2,500 amsl. (2011–2021)

**Table 3:**
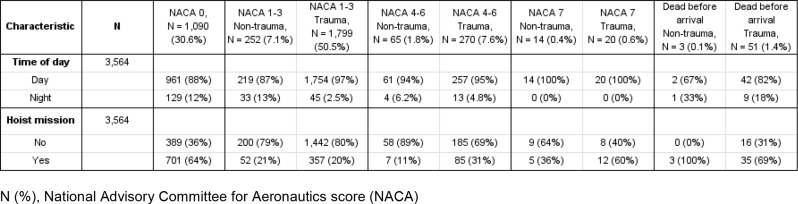
Baseline data on location and altitude of HEMS missions conducted above 2,500 Amsl. (2011–2021)

**Fig. 2:**
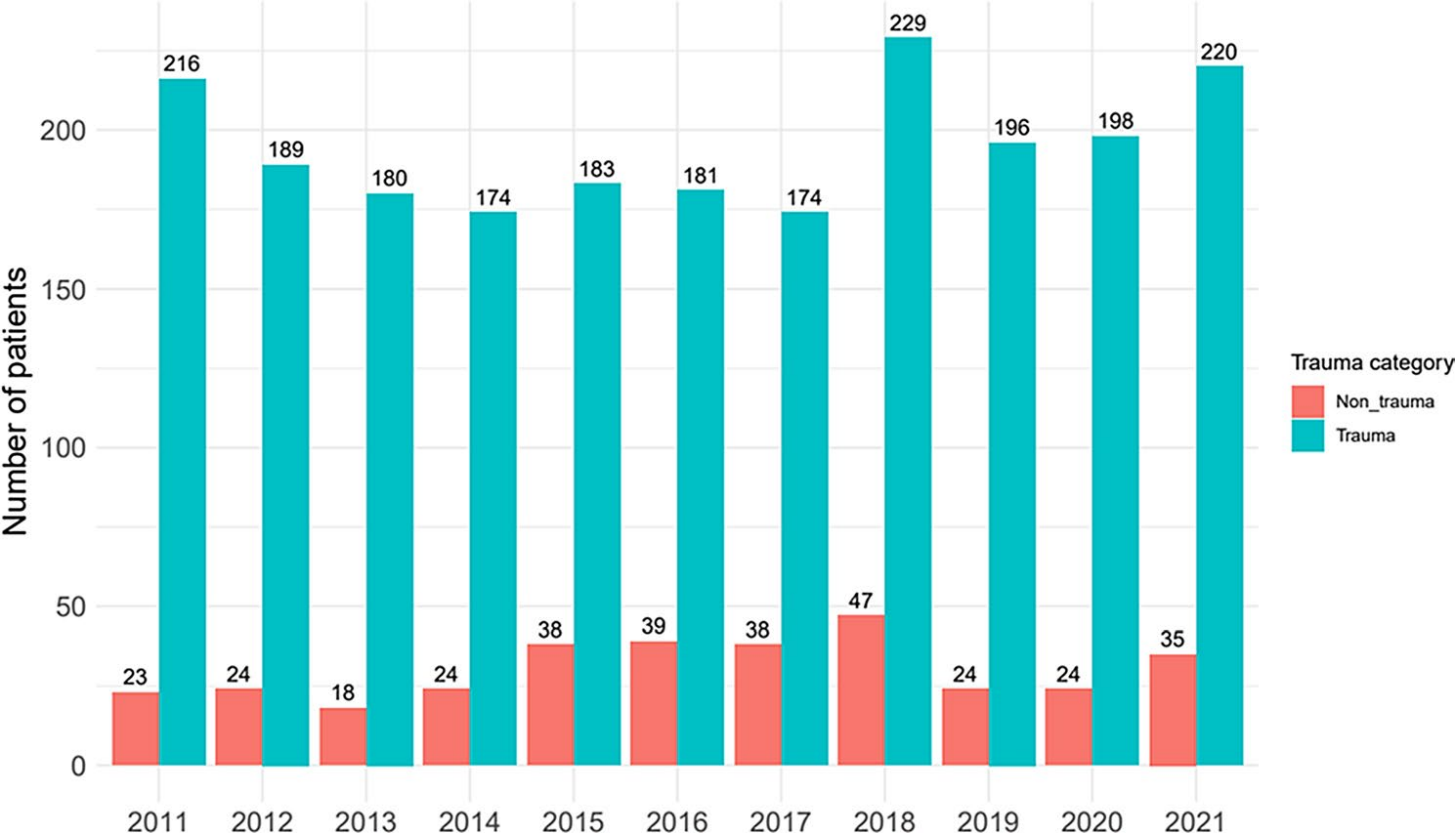
HEMS missions per year divided into trauma and non-trauma missions (2011–2021), *N* = 3,564 patients. Green = trauma missions, red = non-trauma missions

Figure 2 provides an overview of the total number of missions per year in the study period (2011–2021), broken down into trauma and non-trauma deployments. Non-trauma missions represented a minority across all the years (< 20% of annual missions at altitudes > 2,500 amsl.)

**Fig. 3:**
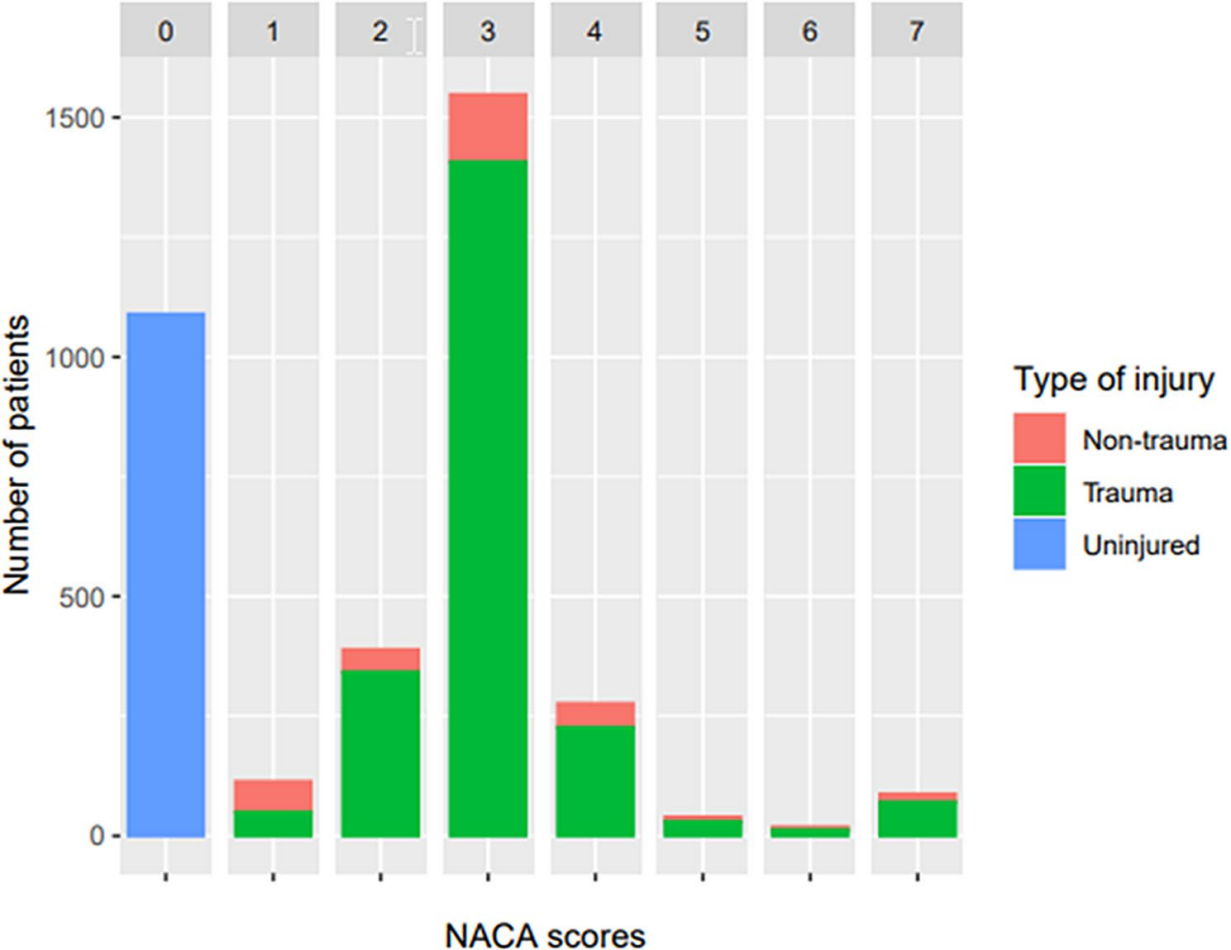
Distribution of rescue missions based on the NACA score, divided into trauma, non-trauma, and uninjured, *N* = 3,564 patients. Red = non-trauma, green = trauma, blue = uninjured

Of the patients treated, 66.8% were male and the majority (84.8%) were adults. Most interventions occurred in remote areas (89.1%), with the majority of missions involving uninjured patients (NACA 0, 30.6%) or minor injured patients (NACA 1–3, 57.6%). Furthermore, 1,257 missions (35.3%) were hoist missions, with uninjured patients (NACA 0) requiring rescue by hoist most frequently (19.6% of all hoist missions). Patients with serious injuries represented a minority (9.4%). Patients who died during medical care represented 1% of the total number of patients. Patients who had already died on arrival make up 1.5% of the total. The distribution of missions among the NACA groups is shown in Fig. 3.

The basic interventions undertaken during the missions, broken down into the individual NACA groups, are shown in Table 1 in the Appendix. It can be seen that significantly more basic measures, such as the insertion of an intravenous line for analgesia, had to be carried out on scene in severely injured patients.

**Fig. 4:**
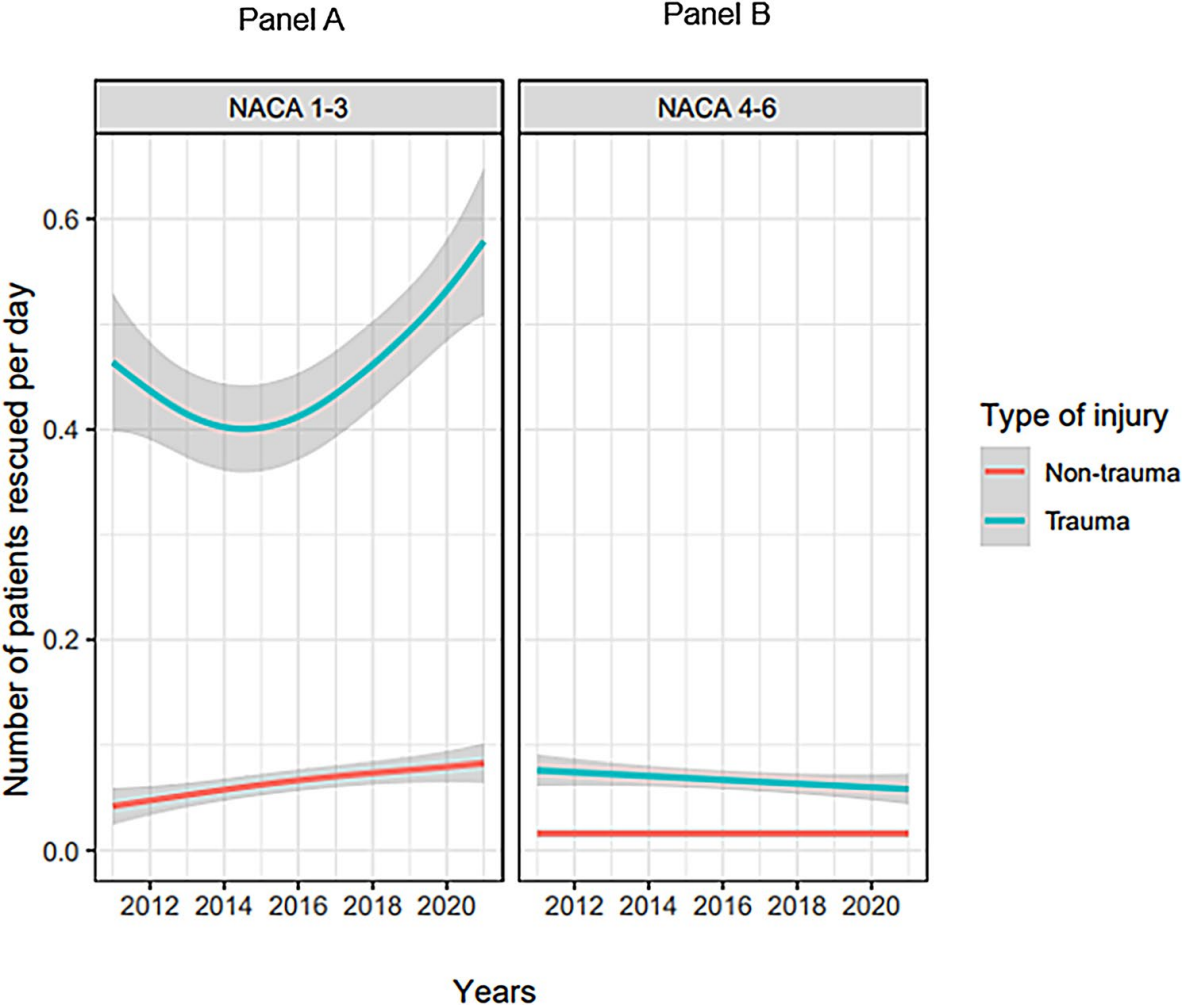
Panel **A** and **B**: Illustration of the number of rescued patients over the observation period of 10 years. *N* = 3,564 patients. Trauma patients (blue), non-trauma patients (red), grey = confidence interval. Panel A: NACA 1–3 patients; panel B: NACA 4–6 patients

Fig. 4 illustrates the missions over the 10-year study period.

### On-scene time and influencing factors

A significant correlation with on-scene time was seen for the NACA score (*p* < 0.001), the use of a hoist (*p* < 0.001), and for trauma patients in summer (*p* = 0.003). Location (*p* = 0.3) and day vs. night (*p* = 0.59) had no significant effect on the on-scene time (see Table 3 in the Appendix).

**Fig. 5:**
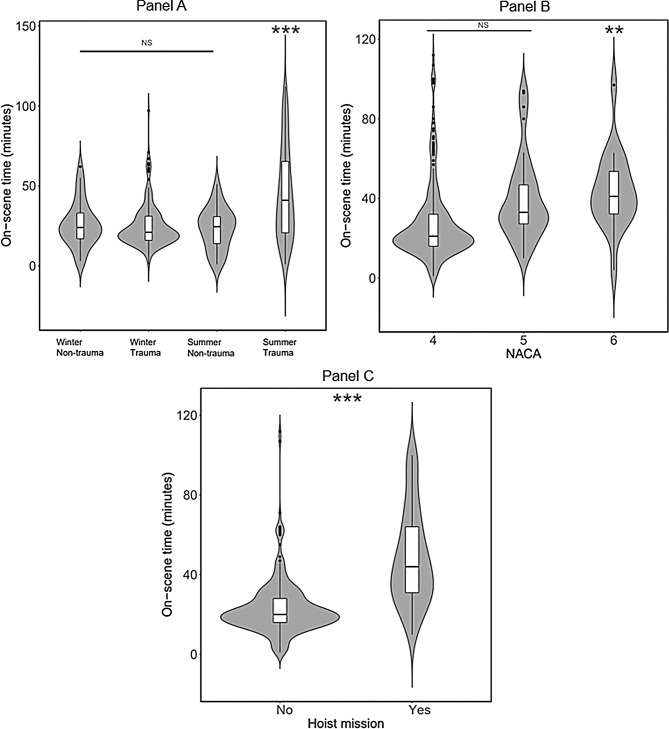
Panel **A**,**B**, and **C**: Box-plot illustration of the significant factors influencing on-scene time. Panel A: Influence of the combined factors of season and trauma diagnosis; panel B: Influence of the NACA score; panel C: Influence of the use of a hoist

The mean on-scene time was longer for NACA 6, at 54.9 min (95% CI [45, 64.9]), compared to NACA 5 (*p* < 0.003) and NACA 4 (*p* < 0.001). No difference in mean on-scene time was found between NACA 4, at 32.8 min (95% CI [26.1, 39.5]), and NACA 5, with a mean on-scene time of 38.5 min (95% CI [30.1, 46.8]), *p* = 0.13 (Fig. 5, panel B). The use of a hoist on patients with NACA 4–6 extended the mean on-scene time from 31.3 min to 52.9 min (95% CI [24.3, 38.2 versus 45, 60.8]), *p* < 0.001 (Fig. 5, panel C; Fig. 1 in Appendix).

As can be seen in Fig. 5, panel A, there is a significant increase in on-scene time for trauma patients in summer compared to trauma patients in winter (*p* < 0.0001), from a mean of 38.2 min (95% CI [30.7, 45.6]) to a mean of 46 min (95% CI [38.6, 53.3]). The independent influencing factors accident type (trauma vs. non-trauma) and time of year did not have a significant influence on the on-scene time (Fig. 5, panel A).

## Discussion

Our data from 3,564 patients in HEMS missions conducted above 2,500 amsl. in Switzerland shows a significant increase in the number of operations involving patients with minor injuries in recent years. Moreover, the on-scene time for patients with NACA scores of 4–6 was influenced by the NACA score, the use of a hoist, and trauma diagnoses in summer.

### Patient population and injury severity

In line with the existing literature, our baseline data, presented in Tables 1, 2 and 3, showed that most rescued patients were adult men engaged in summer sport activities [9, 10]. Also in line with previous studies, our data showed a significantly higher number of trauma patients than medical patients in high alpine regions [5]. Moreover, regardless of the severity of the injury, most of the missions took place in remote areas, and most of these missions did not require a hoist. The severity of the injury or illness that we were able to identify in our data is comparable with the current data in the literature [6, 10, 11]. Finally, we found that, as in comparable studies, the number of rescue missions has increased in recent years, while the severity of the injuries has decreased [10].

One reason for the increase in the number of accidents in high alpine areas is undoubtedly the increase in the number of people engaging in leisure activities in high alpine terrain [1–3]. However, risk-taking behavior, poor physical conditioning, lack of preparation, and underestimating the altitude and terrain are likely contributing factors to the increasing frequency of injuries during mountain hiking and rock climbing. Limited safety awareness has been more commonly observed in middle-aged men [12]. A recent study of paragliders who were rescued by helicopter showed that too little experience and too many errors of judgment are the most common factors contributing to incidents [13].

At this point, we should also consider whether air rescue missions are prioritizing comfort medicine over life-saving medicine. In 2006, Kaufmann et al. described a trend towards comfort medicine in their analysis conducted in Innsbruck, Austria [10]. They noted an increasing number of missions with a decreasing average NACA score. The growing numbers of medically non-indicated calls can lead to greater dissatisfaction and boreout among HEMS personnel. At the same time, many uninjured patients (NACA 0) are proactively evacuated by HEMS teams, giving these HEMS missions a preventive character, as they prevent those affected from being injured in the end. However, the real risk of accidents involving medical helicopters must also be considered when transporting patients with minor injuries by HEMS [14]. The risk of accidents is greater for HEMS than for ground services, which must be taken into consideration whenever a HEMS is requested. It is true, however, that even minor injuries at a high altitude and in difficult terrain may become potentially life threatening and require HEMS assistance if the patients cannot be safely rescued by other means.

### HEMS dispatch practice and the potential for future triage optimization

Our findings must be interpreted within the context of the Swiss emergency rescue system, which -like those in other alpine regions- is shaped by unique geographical and structural challenges. In high mountain terrain above 2500 m, HEMS dispatch decisions are often driven more by topographical inaccessibility than by the patient’s clinical condition. Many missions involve individuals with minor or no injuries who are stranded in remote or hazardous locations where ground-based evacuation would be slow, logistically complex, or unsafe. In such scenarios, air rescue serves a “preventive” function, averting deterioration due to environmental exposure, hypothermia, exhaustion, or secondary accidents during attempted self-rescue. While this approach ensures patient safety, the rising number of low-severity cases raises important questions about resource allocation, cost-effectiveness, and environmental sustainability. Exploring alternative models, such as expanding the role of non-medical mountain rescue services, introducing intermediate response tiers, or refining triage protocols, could help maintain rapid access to remote areas while preserving HEMS capacity for truly time-critical, life-saving missions.

### Expertise spectrum of the HEMS team

9% of the rescued patients were seriously injured and complex medical measures had to be implemented on scene. In 2019, Pietsch et al. analyzed more than 10,000 rescue missions in Switzerland and showed how important it is to have well-trained medical personnel on board, who are able to carry out complex medical interventions under difficult circumstances [5]. The fact that complex rescues missions are not an everyday occurrence is the reason why it is so important for procedures to be constantly practiced, so that they can be flawlessly executed in the event of an emergency. Regular simulation training has been shown to improve team resource management and can have a positive impact on decision making and patient outcomes [15]. Given the specialized nature of air rescue operations, it is crucial that all crew members are thoroughly trained, both medically and in terms of the environment.

The Swiss Air Rescue organization has clear guidelines for its helicopter emergency doctors [16], ensuring that medical staff meet the necessary qualifications and competencies.

Additionally, Rega offers annual internal simulation training and partner days with Alpine Rescue Switzerland, to maintain readiness and foster collaboration with mountain rescuers [15].

### Impact of on-scene time

In line with the results of Fuchs et al. [17], we were able to show using our data that there was a significant increase in on-scene time with increasing NACA score and the associated increase in measures on-scene. Previous studies have shown that prolonged on-scene times are significantly associated with increased mortality of trauma patients. This highlights the importance of identifying modifiable factors that contribute to on-scene time in order to support better patient outcomes. It is important to acknowledge, however, that patients with higher NACA scores typically present with more severe injuries, necessitating more intensive on-scene interventions. As a result, longer on-scene times and higher mortality may both reflect the severity of the condition, introducing a potential confounding factor or bias in this association [18].

It is well known that hoist operations significantly increase time on scene, something also seen in our data. However, they are often the only way to access the patient, especially in very rough terrain [17, 19].

The association between trauma events in summer and longer on-scene time may be influenced by several factors. One possible explanation is that trauma events in winter often occur on ski slopes, where patients can be given first aid by the ski patrol service, leading to shorter on-scene times. However, higher ambient temperatures in summer may contribute to an increase in the number of interventions, especially for trauma patients, that are performed on scene, rather than in the cabin or even at the destination hospital.

### Limitations

Our study has several limitations, beginning with its retrospective nature, which may contribute to statistical bias (e.g., incomplete or invalid documentation, characteristics that cannot be clearly assigned). We believe that these data are missing at random and therefore should not have biased our results. Another limitation is the lack of hospital and follow-up data, as well as long-term clinical outcomes, although this does not affect the main focus of this study. Finally, although the NACA score shows a strong correlation with the patient’s vital risk and good interrater reliability, it still has its limitations [20, 21]. Nevertheless, it would be valuable to explore several aspects, such as whether preclinical diagnoses are confirmed in the hospital, which time-consuming medical interventions could be effectively carried out in-flight to minimize on-scene time, and whether preclinical injury management can predict long-term patient outcomes.

## Conclusion

The data indicates that the number of alpine rescue missions undertaken for patients with minor to moderate injuries above 2,500 amsl. has increased over the past decade. More than half of these missions involved treating minor injured patients, while the large number of HEMS missions with uninjured patients had a preventive character. Meanwhile, roughly 9% of the patients evacuated were critically injured, requiring complex, life-saving medical interventions. Hoist missions, seriously injured patients, and missions with trauma patients in summer were correlated with a significant increase in on-scene time.

## Electronic supplementary material

Below is the link to the electronic supplementary material.

Supplementary Material 1: Additional file 1: Appendix with additional tables and figures as a Word document (.doc)

## Data Availability

No datasets were generated or analysed during the current study.

## Abbreviations

AMSL: Above mean sea level
ARS: Alpine Rettung Schweiz
CPR: Cardiopulmonary resuscitation
ECG: Electrocardiogram
HEMS: Helicopter Emergency Medical Service
IFR: Instrument flight rules
KED: Kendrick extrication device
NACA: National Advisory Committee for Aeronautics
NIV: Non-invasive ventilation
NVG: Night vision goggles
REGA: Swiss Air Ambulance
RSI: Rapid Sequence Induction
VFR: Visual flight rules
