# Supplementary material for: High-altitude HEMS missions—a retrospective analysis of 3,564 air rescue missions conducted between 2011 and 2021
Source: Scand J Trauma Resusc Emerg Med. 2025 May 30;33:97. doi: 10.1186/s13049-025-01419-x (PMC12123734; doi:10.1186/s13049-025-01419-x)
Supplement: Supplementary file 1 — Supplementary Material 1: Additional file 1: Appendix with additional tables and figures as a Word document (.doc) [file 13049_2025_1419_MOESM1_ESM.docx]

**Appendix**

Table 1

**Table 1: Basic interventions during missions.**


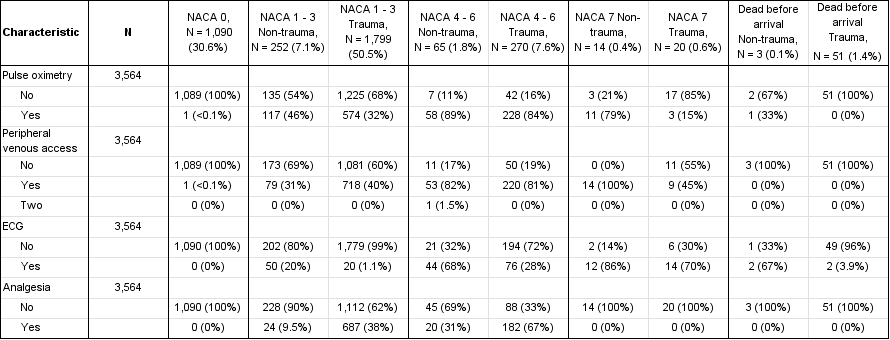


N (%), Electrocardiogram (ECG), Analgesia: Morphine, Fentanyl, Ketamine

Table 2

**Table 2: Additional measurements during missions**


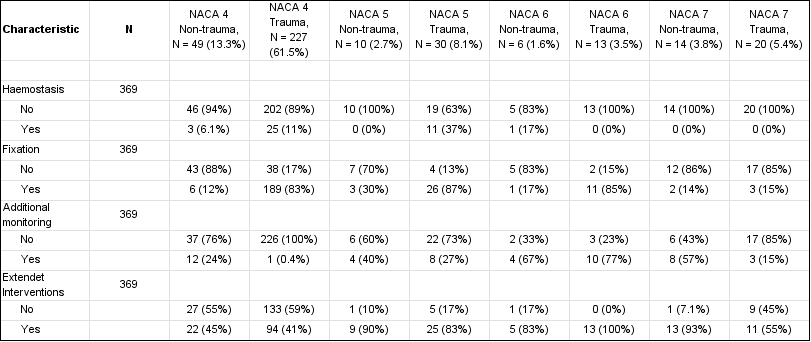


N (%). Haemostasis (Tourniquet bandage, application of i.v. tranexamic acid); Fixation (cervical spine fixation, scoop stretcher, spinal board, kendrick extrication device (KED), vacuum mattress, pelvic sling, splint-fixation (e.g. SamSplint); Additional monitoring (12-lead electrocardiogram (ECG), capnography, telemetry); Extended interventions (Oxygen administration, non-invasive ventilation (NIV), cardiopulmonary resuscitation (CPR), defibrillation, intubation, coniotomy, chest decompression).

Table 3

**Table 3: Influence of selected factors on on-scene time**


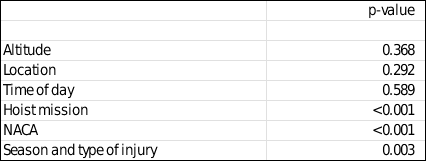


Location = remote vs. accessible, Time of day = day vs. night, Hoist mission = yes vs. no,
NACA = NACA 4 vs. NACA 5 vs. NACA 6, Season in connection with type of injury = trauma vs. non-trauma)

Figure 1


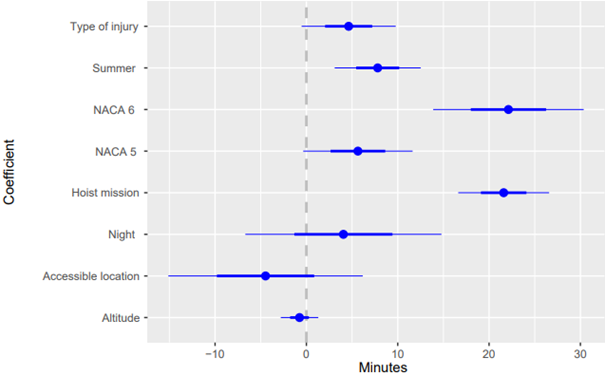


**Fig. 1: Coefficient plot to visualize the results of the linear regression of various factors influencing the on-scene time.** The plot shows the change in on-scene time compared to a reference. Reference NACA score = NACA 4, reference hoist mission = no hoist used, reference trauma = non-trauma, reference summer = winter, reference night = day, reference accessible = remote
